# Complete remission of rare adenocarcinoma of the oropharynx with APCEDEN ^®^ (dendritic cell‐based vaccine): a case report

**DOI:** 10.1002/ccr3.1169

**Published:** 2017-09-07

**Authors:** Chaitanya Kumar, Sakshi Kohli, Srikanth Chiliveru, Minish Jain, Bandana Sharan

**Affiliations:** ^1^ R&D APAC Biotech Pvt Ltd Gurgaon India; ^2^ Medical Oncology Ruby Hall Clinic Pune India

**Keywords:** APCEDEN, dendritic cells, immunotherapy, oropharyngeal carcinoma, retromolar trigone

## Abstract

APCEDEN ^®^ is an autologous monocyte‐derived dendritic cell‐based immunotherapy. A 58‐year‐old man with adenocarcinoma of oropharynx shows complete remission after receiving APCEDEN ^®^ in conjunction with Geftinib validated by reduction in size, whereas Gefitinib alone lead to disease progression.

## Introduction

Oropharyngeal Carcinoma is one of the most common head and neck cancers. Over 90% of the oropharyngeal cancers are squamous cell carcinoma in origin with a yet undetermined percentage being adenocarcinoma 1, 2. Adenocarcinoma is a common malignancy emerging from minor salivary glands, and in other organs such as lung, prostate, pancreas, colon, esophagus, and the oropharyngeal area. The most common region for oropharyngeal adenocarcinoma is the superficial lobe of the parotid gland, followed by the deep lobes and usually never identified in the retromolar trigone area (RMT) 2, 3. Cancerous lesions in the retromolar trigone space are almost always squamous cell carcinomas, thus make this case rare 4, 5, 6.

Dendritic cells (DC) are the professional antigen presenting cells of the immune system with a capability to ingest and process antigens in the peripheral blood and tissues. They can migrate to the lymph nodes and present antigens to lymphocytes, thus being a bridge between innate and adaptive immune response 7. Dendritic cells in their immature state are good in taking up antigens which leads to maturation of DCs to cells with increased capacity for antigen presentation and T‐cell activation 8. A DC vaccine involves loading of tumor antigens on immature DCs, which after infusion into patients induce a T‐cell response against tumor cells expressing those antigens. DCs constitute a very small percentage of white blood cells; hence, DCs are harvested in the laboratory from peripheral blood mononuclear cells (PBMCs) using differentiation stimuli cytokines. Antigenic cargo loaded onto DCs can be from various sources and is very important in determining the success of the vaccine. Endogenous and exogenous antigens are processed differently and bind to different MHC molecules thereby altering the types of T cells activated 9.

Dendritic cells immunotherapy has been shown to be feasible, effective and safe against various kinds of cancer by many research groups 10, 11, 12. MUC1 and MAGE‐3 peptide‐pulsed DCs are shown to be effective against lung adenocarcinoma 13. Cytotoxic T‐lymphocyte (CTL) response after DC vaccination was observed in a group of patients with glioblastoma 14. Bapsy et al., 2012, presented a phase II study to check the efficacy of APCEDEN^®^ in various cancer indications with a total of 51 subjects. They reported an objective response rate of 28.9% by RECIST and 42.1% by irRC along with 9 weeks of median time to progression 15. APCEDEN^®^ therapy also provides a survival benefit of approximately 200 days over the control groups (publication in review). DC vaccination alone or in combination with other drugs like immune checkpoint inhibitors opens a new promising future to the patients with cancer 16. In this study, we present a patient with adenocarcinoma of the oropharynx who received chemotherapy and radiation therapy as first line of treatment followed by APCEDEN^®^ that led to disease control and a complete response by tumor size assessment.

## APCEDEN^®^ Preparation

The process begins with isolating PBMCs from the patient by apheresis and isolating monocytes by plastic adherence. Further differentiation to DCs is carried out by culturing the monocytes in media supplemented with IL‐4 and GM‐CSF for 5 days. On the sixth day, DCs are co‐incubated with the tumor lysate prepared from the tissue biopsy sample of the patient followed by the maturation stimuli. Mature DCs harvested on the 8th day undergo a quality check for the phenotypic markers. Viability of DCs is assessed by 7AAD staining. Further, the vaccine is also tested for sterility, mycoplasma, and endotoxin contamination. Six doses (4–5 million mature DCs in each dose) in a time frame of 14 weeks are given to the patient via intravenous route. Lack of sufficient tumor tissue from patient biopsies, unable to isolate the required PBMC population, clinical situations in which tumors considered inoperable and unreachable, invasiveness in biopsy/surgical procedure damaging the quality of life, patients with multiple tumors, and stage of the cancer in conjunction with the age of the patient are some of the limitations of the procedure.

## Case History

A 58‐year‐old gentleman from Pune, India, experienced swelling in the left submandibular region for 6 months and was diagnosed with adenocarcinoma of the oropharynx in June 2010 but did not receive any adjuvant treatment. MR scan of the neck revealed a mass of 4.4 × 4.2 × 5.8 cm in transverse, anteroposterior, and superoinferior dimensions that extends laterally into the left parapharyngeal space. The left level II lymph node was enlarged measuring 3.4 × 3.6 cm. Tumor was a large lobulated enhancing mass lesion involving the base of the tongue and faucial tonsil on the left side extending in the left vallecula, oropharynx, pre‐epiglottic space, parapharyngeal space, and retromolar trigonal space on the left side with extension across the midline. Superiorly, it extended up to the left fossa of rosenmuller. CT scan of neck disclosed a large locally infiltrative oropharyngeal neoplastic mass on the left jugulodigastric lymph node involving tongue base/tonsil/lateral oropharyngeal wall with likely extension to oral tongue/floor of the mouth/nasopharynx.

**Figure 1:**
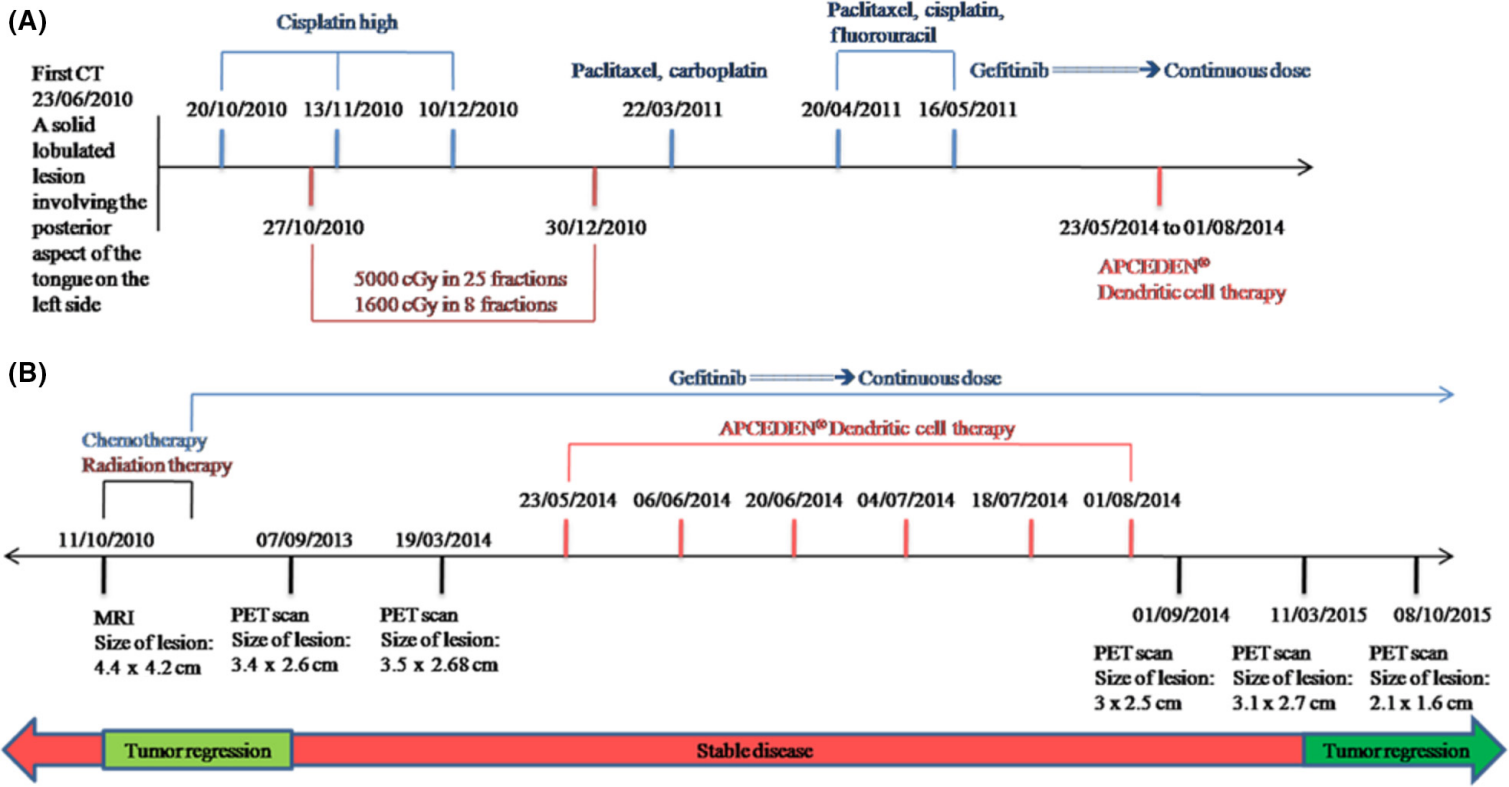
(A) The course for the treatment given from the date of diagnosis. (B) Treatment regime with concomitant PET/CT scans to show disease course.

The patient was subjected to chemotherapy and radiation therapy for 7 and 2 months, respectively. The course for the treatment is explained in Figure 1A. He received Inj. Cisplatin (100 mg/m^2^) for first three rounds of chemotherapy followed by Inj. paclitaxel (175 mg/m^2^) and carboplatin (AUC5) for the fourth cycle. Combination of paclitaxel (175 mg/m^2^), cisplatin (25 mg/m^2^) and fluorouracil (750 mg/m^2^) was administered for the last two sessions of chemotherapy. He also received concurrent radiation therapy of total 6600 cGy in 33 fractions during the first session of chemotherapy. There was an initial tumor regression (3.4 × 2.6 cm) followed by a stable disease upon Gefitinib treatment.

**Figure 2:**
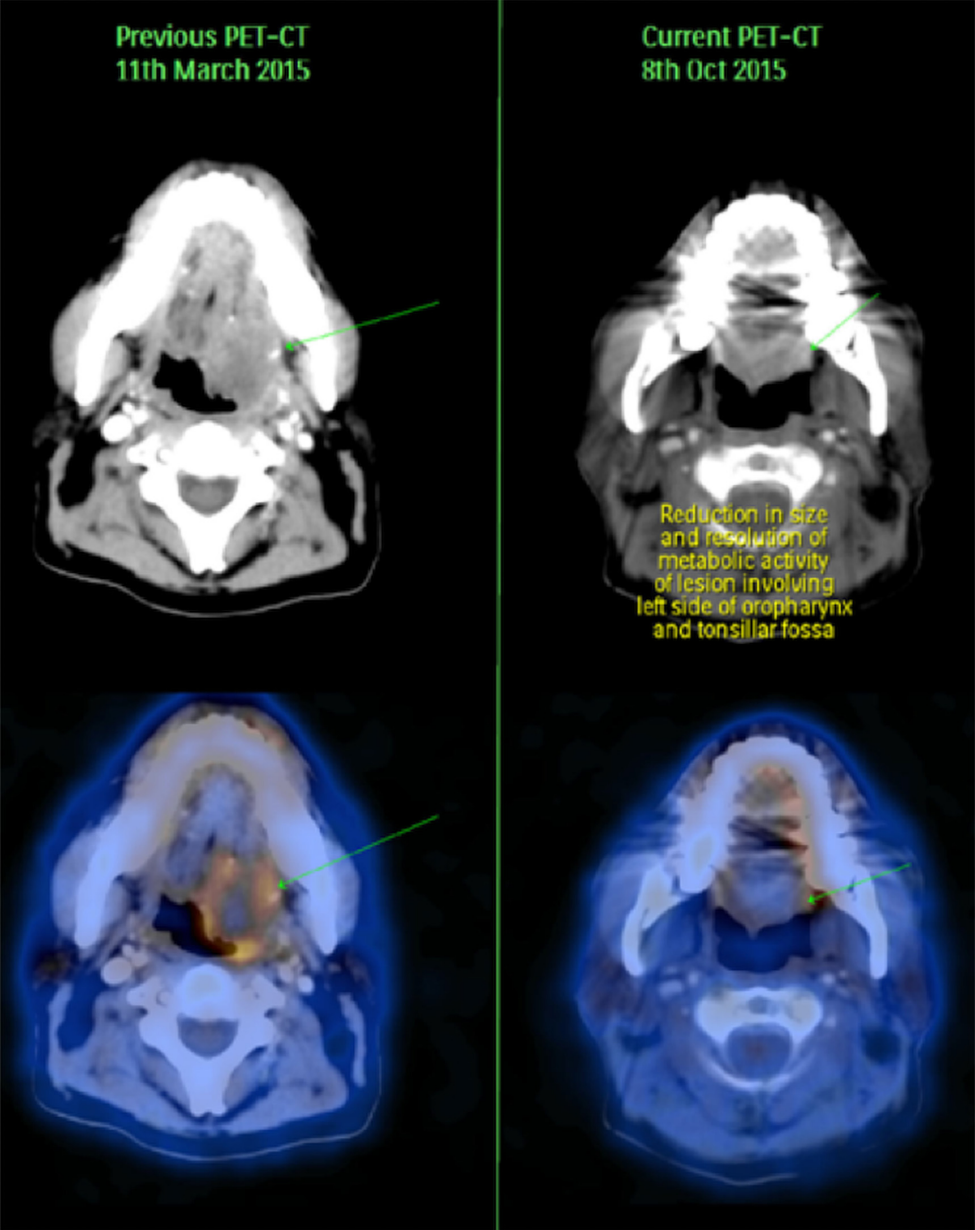
Comparison of PET‐CT scan of the subject conducted on 11/03/2015 (~8 months after last dose of APCEDEN) with PET‐CT scan on 08/10/2015 (~14 months after last dose of APCEDEN). Reduction in tumor size is indicated by green arrow in the image.

Due to no further reduction in the tumor size even after continuous dose of Gefitinib for 3 years, treatment using APCEDEN^®^ was initiated on 14 May 2014. Six doses of DC vaccine were administered between 23/05/2014 and 01/08/2014 with parallel Gefitinib medication. PET scans in October 2015 highlighted a dramatic decrease in the tumor size, 2.1 × 1.6 cm versus 3.1 × 2.7 cm as observed in March 2015 (Fig. 2). The treatment regime with serial PET/CT scans showing the disease course is presented in Figure 1B.

Hemogram report of the patient at baseline before the immunotherapy and two vital stages of the therapy (3rd and 6th dose) reveal a progressive effect of the therapy by enumerating the neutrophil lymphocyte ratio (NLR) of the pathological test results. NLR is considered as an independent prognostic factor for variety of cancers 17, 18 and also a robust early response biomarker of therapeutic efficacy 19. Table 1 shows a decreasing trend in the ratio which is expected in a functional therapy.

**Table 1:** Neutrophil lymphocyte ratio (NLR) of the patient at various stages of APCEDEN^®^ therapy

	Baseline	3rd Dose	6th Dose
Neutrophil	18.4	20.6	20.6
Lymphocyte	68.9	65.9	21.0
NLR	3.74	3.19	2.99

## Discussion

Over the past few decades, there has been a shift in conventional therapies for cancer with immunotherapy booming as a promising candidate with better quality of life and survival benefits for cancer patients 20. In India, oral cancer is the commonest of head and neck cancer out of which adenocarcinoma of the oropharynx constitutes a minor percentage. Here, we describe a patient diagnosed with adenocarcinoma of the oropharynx, and the complete response was achieved after DC‐based immunotherapy. Administration of six cycles of chemotherapy and concurrent radiation therapy resulted in initial tumor size reduction but led to a stable disease thereafter. Gefitinib is a signal transduction inhibitor which targets epidermal growth factor receptor (EGFR) tyrosine kinase. Tyrosine Kinase inhibitors are approved for use in various cancer types; however, it is common that patient's tumor develop resistance to them. This might explain the disease progression after 3 years of Gefitinib administration for the patient mentioned in this report, which made the treating doctor add DC therapy to the Gefitinib administration.

Personalized vaccines for cancer are shown to be effective in many cancer types. APCEDEN^®^ that contains patients’ own DCs pulsed with the patient‐specific tumor antigens has shown to be efficient in other cancer types. In this case also, we observed tumor regression after 14 months of receiving DC therapy. DC vaccines are demonstrated to be nontoxic by various groups 21, 22. APCEDEN^®^ was well tolerated by the patient in this case with no adverse events reported.

Skewing the immune system in favor of the patient involves fighting through the immune‐suppressive environment created by the tumor cells and activating the host defenses against tumor. Augmenting the potential of our immune system to fight cancer using DCs involves activation of multiple immune cells such as CD4+, CD8+, and macrophages. As DC vaccine is a form of active immunotherapy that engages our immune system to fight against tumor, the time taken to actually see tumor reducing after DC therapy can be long and vary from patient to patient 23. This was concordant with the decrease in tumor size (3.0 × 2.5 cm) after 1 month of receiving last dose of APCEDEN^®^ followed by slight increase in tumor mass (3.1 × 2.7 cm) before final regression (2.1 × 1.6 cm) after 14 months (Fig. 1B). Increase in tumor mass reported after few months of immunotherapy might be attributed to the infiltrating immune cells at the tumor site 24, 25.

The patients with baseline NLR < 5 had a significantly improved progression‐free survival (PFS) and overall survival (OS) compared with those with NLR ≥ 5. Associations of low NLR with improved survival are confirmed in various validation cohorts of patients 26. In agreement with the published reports, the patients displayed a decreasing NLR < 5 supporting our results showing complete remission of adenocarcinoma in the present case study.

As this is a rare form of cancer, and no standard treatment modalities are in practice, the DC therapy that can be administered simultaneously along with Gefitinib medication is carried out in the present case. As the patient was aged and keeping in view the stage of the carcinoma, we could only enumerate limited clinical and pathological parameters which are presented in the case study. Further assessment of the subject to determine the tumor‐free status and long‐lasting immunity to prevent relapse is required.

## Conflict of Interest

None declared.

## Authorship

CK, BS, and SK: performed the dendritic cell therapy (APCEDEN^®^), wrote the original manuscript. MJ: performed chemotherapy and radiation, did infusion of APCEDEN^®^, and obtained the patients consent. CK, BS, and SC: reviewed and edited the final manuscript.

## References

[1] Majchrzak, E. , B. Szybiak , A. Wegner , P. Pienkowski , J. Pazdrowski , L. Luczewski , et al. 2014 Oral cavity and oropharyngeal squamous cell carcinoma in young adults: a review of the literature. Radiol. Oncol. 48:1–10. 24587773 10.2478/raon-2013-0057 PMC3908841

[2] Cohan, D. M. , S. Popat , S. E. Kaplan , N. Rigual , T. Loree , and W. L. Hicks Jr . 2009 Oropharyngeal cancer: current understanding and management. Curr. Opin. Otolaryngol. Head Neck Surg. 17:88–94. 19373958 10.1097/moo.0b013e32832984c0

[3] Chi, A. C. , T. A. Day , and B. W. Neville . 2015 Oral cavity and oropharyngeal squamous cell carcinoma—an update. CA Cancer J. Clin. 65:401–421. 26215712 10.3322/caac.21293

[4] Huang, C. J. , K. S. Chao , J. Tsai , J. R. Simpson , B. Haughey , G. J. Spector , et al. 2001 Cancer of retromolar trigone: long‐term radiation therapy outcome. Head Neck 23:758–763. 11505486 10.1002/hed.1108

[5] Rachel, J. R. , N. S. Kumar , and N. K. Jain . 2011 Basaloid squamous cell carcinoma of retromolar trigone: a case report with review of literature. J. Oral Maxillofac. Pathol. 15:192–196. 22529579 10.4103/0973-029X.84495 PMC3329705

[6] Faisal, M. , T. Abbas , U. Khaleeq , M. Adeel , A. W. Anwer , R. Hussain , et al. 2017 Treatment outcomes of rare retromolar trigone squamous cell carcinoma using combined modalities. Cureus 9:e1203. 28580200 10.7759/cureus.1203 PMC5451270

[7] Figdor, C. G. , I. J. de Vries , W. J. Lesterhuis , and C. J. Melief . 2004 Dendritic cell immunotherapy: mapping the way. Nat. Med. 10:475–480. 15122249 10.1038/nm1039

[8] O'Neill, D. W. , S. Adams , and N. Bhardwaj . 2004 Manipulating dendritic cell biology for the active immunotherapy of cancer. Blood 104:2235–2246. 15231572 10.1182/blood-2003-12-4392

[9] Ackerman, A. L. , C. Kyritsis , R. Tampe , and P. Cresswell . 2003 Early phagosomes in dendritic cells form a cellular compartment sufficient for cross presentation of exogenous antigens. Proc. Natl. Acad. Sci. U S A 100:12889–12894. 14561893 10.1073/pnas.1735556100 PMC240714

[10] Dhodapkar, M. V. , R. M. Steinman , M. Sapp , H. Desai , C. Fossella , J. Krasovsky , et al. 1999 Rapid generation of broad T‐cell immunity in humans after a single injection of mature dendritic cells. J. Clin. Invest. 104:173–180. 10411546 10.1172/JCI6909 PMC408478

[11] Mackensen, A. , B. Herbst , J. L. Chen , G. Kohler , C. Noppen , W. Herr , et al. 2000 Phase I study in melanoma patients of a vaccine with peptide‐pulsed dendritic cells generated in vitro from CD34 (+) hematopoietic progenitor cells. Int. J. Cancer 86:385–392. 10760827 10.1002/(sici)1097-0215(20000501)86:3<385::aid-ijc13>3.0.co;2-t

[12] Banchereau, J. , A. K. Palucka , M. Dhodapkar , S. Burkeholder , N. Taquet , A. Rolland , et al. 2001 Immune and clinical responses in patients with metastatic melanoma to CD34 (+) progenitor‐derived dendritic cell vaccine. Cancer Res. 61:6451–6458. 11522640

[13] Wojas‐Krawczyk, K. , P. Krawczyk , J. Buczkowski , A. Walkowska , O. Jankowska , E. Czekajska‐Chehab , et al. 2012 Immunotherapy of lung adenocarcinoma patient with Peptide‐pulsed dendritic cells: a case report. Arch. Immunol. Ther. Exp. (Warsz) 60:69–77. 22143160 10.1007/s00005-011-0157-7

[14] Liau, L. M. , R. M. Prins , S. M. Kiertscher , S. K. Odesa , T. J. Kremen , A. J. Giovannone , et al. 2005 Dendritic cell vaccination in glioblastoma patients induces systemic and intracranial T‐cell responses modulated by the local central nervous system tumor microenvironment. Clin. Cancer Res. 11:5515–5525. 16061868 10.1158/1078-0432.CCR-05-0464

[15] Bapsy, P. P. , B. Sharan , C. Kumar , R. P. Das , B. Rangarajan , M. Jain , et al. 2014 Open‐label, multi‐center, non‐randomized, single‐arm study to evaluate the safety and efficacy of dendritic cell immunotherapy in patients with refractory solid malignancies, on supportive care. Cytotherapy 16:234–244. 24438902 10.1016/j.jcyt.2013.11.013

[16] Halpert, M. M. , V. Konduri , D. Liang , Y. Chen , J. B. Wing , S. Paust , et al. 2016 Dendritic cell‐secreted cytotoxic T‐lymphocyte‐associated protein‐4 regulates the T‐cell response by downmodulating bystander surface B7. Stem Cells Dev. 25:774–787. 26979751 10.1089/scd.2016.0009 PMC4870609

[17] Faria, S. S. , P. C. Fernandes Jr , M. J. B. Silva , V. C. Lima , W. Fontes , R. Freitas‐Junior , et al. 2016 The neutrophil‐to‐lymphocyte ratio: a narrative review. Ecancermedicalscience 10:702. 28105073 10.3332/ecancer.2016.702 PMC5221645

[18] Piciucchi, M. , S. Stigliano , L. Archibugi , G. Zerboni , M. Signoretti , V. Barucca , et al. 2017 The neutrophil/lymphocyte ratio at diagnosis is significantly associated with survival in metastatic pancreatic cancer patients. Int. J. Mol. Sci. 18:730. 10.3390/ijms18040730 PMC5412316 28353661

[19] Templeton, A. J. , J. J. Knox , X. Lin , R. Simantov , W. Xie , N. Lawrence , et al. 2016 Change in neutrophil‐to‐lymphocyte ratio in response to targeted therapy for metastatic renal cell carcinoma as a prognosticator and biomarker of efficacy. Eur. Urol. 70:358–364. 26924770 10.1016/j.eururo.2016.02.033

[20] Palucka, K. , and J. Banchereau . 2013 Dendritic‐cell‐based therapeutic cancer vaccines. Immunity 39:38–48. 23890062 10.1016/j.immuni.2013.07.004 PMC3788678

[21] Schuler‐Thurner, B. , E. S. Schultz , T. G. Berger , G. Weinlich , S. Ebner , P. Woerl , et al. 2002 Rapid induction of tumor‐specific type 1 T helper cells in metastatic melanoma patients by vaccination with mature, cryopreserved, peptide‐loaded monocyte‐derived dendritic cells. J. Exp. Med. 195:1279–1288. 12021308 10.1084/jem.20012100 PMC2193752

[22] Boudewijns, S. , H. Westdorp , R. H. Koornstra , E. H. Aarntzen , G. Schreibelt , J. H. Creemers , et al. 2016 Immune‐related adverse events of dendritic cell vaccination correlate with immunologic and clinical outcome in stage III and IV melanoma patients. J. Immunother. 39:241–248. 27227325 10.1097/CJI.0000000000000127 PMC4902323

[23] Hoos, A. 2012 Evolution of end points for cancer immunotherapy trials. Ann. Oncol. 23(Suppl. 8):viii47–viii52. 22918928 10.1093/annonc/mds263

[24] Wolchok, J. D. , A. Hoos , S. O'Day , J. S. Weber , O. Hamid , C. Lebbe , et al. 2009 Guidelines for the evaluation of immune therapy activity in solid tumors: immune‐related response criteria. Clin. Cancer Res. 15:7412–7420. 19934295 10.1158/1078-0432.CCR-09-1624

[25] Chiou, V. L. , and M. Burotto . 2015 Pseudoprogression and Immune‐Related Response in Solid Tumors. J. Clin. Oncol. 33:3541–3543. 26261262 10.1200/JCO.2015.61.6870 PMC4622096

[26] Ferrucci, P. F. , S. Gandini , A. Battaglia , S. Alfieri , A. M. Di Giacomo , D. Giannarelli , et al. 2015 Baseline neutrophil‐to‐lymphocyte ratio is associated with outcome of ipilimumab treated Metastatic melanoma patients. Br. J. Cancer 112:1904–1910. 26010413 10.1038/bjc.2015.180 PMC4580390

